# Editorial: Cognition in Parkinson's disease

**DOI:** 10.3389/fcogn.2026.1850845

**Published:** 2026-05-07

**Authors:** Elke Kalbe, Wolfgang H. Jost

**Affiliations:** 1 Medical Psychology | Neuropsychology and Gender Studies and Center for Neuropsychological Diagnostics and Intervention, Faculty of Medicine and University Hospital Cologne, University of Cologne, Cologne, Germany; 2 Parkinson-Klinik Ortenau, Wolfach, Germany

**Keywords:** cognition, cognitive training, dementia, pharmacotherapeutic approaches, prevention

There is still a lack of research on the cognitive abilities of patients with Parkinson's disease. Therefore, this Research Topic is dedicated to the cognitive symptoms in Parkinson's disease. When we were first working on Parkinson's disease in the 1990s, the motor symptoms held the main stage. Even today, our focus is not on non-motor disturbances, which more often are attributed not to the disease itself but rather to other factors; usually, age is brought into play, along with comorbidities and even the patients' medication. This is still the case today when, for example, complaints of constipation, problems in circulation, or fatigue come up. There is a misperception here. Fortunately, there is a somewhat different story in the case of autonomic disturbances: In recent years, this misconception has essentially changed so that coming generations of physicians will start their clinical work realizing that non-motor problems are, in fact, frequent and very relevant in Parkinson's. However, how does this principle also apply to neuropsychiatric symptoms and deficits?. Are depression and hallucinations directly associated with the primary disease itself or the medical therapy? An even more pertinent question is that of cognitive disorders: *Specific cognitive deficits can seriously hamper a patient's chances of coping well with Parkinson's*. An essential question is whether cognitive performance is routinely evaluated. Is the cognitive evaluation rather differentiated or only an approximate indication? Does the evaluation make use of specific cognitive tests or merely non-specific ones, and have they been standardized? Diagnosticians preferentially apply the tests they have been using for years, which may very well mean that they do not make use of the fully standardized neuropsychological testing procedures that are routine in clinics specializing in Parkinson's disease.

A further issue is that we do not yet have a convincing pharmacological therapy for these cognitive deficits. Rivastigmine has been approved here to treat Parkinson's disease-related dementia, but its therapeutic success is not yet fully convincing. Furthermore, pharmacological interventions to treat mild cognitive impairment in subjects with Parkinson's disease or prevent cognitive decline are lacking. Importantly, we definitely do not want to incite serious concerns or even alarm. When, on the one hand, good therapies are lacking, one is tempted to avoid directly asking the patients questions about that precise topic. On the other hand, it is also undeniable that better therapies are not even sought after when the medical need fails to be considered or is considered as secondary. Of course, we want to avoid causing any of our patients anxiety or stigmatizing them. However, if cognitive deficits are not taken on aggressively and if they are oversimplified and only attributed to age or medication, or if the issue is assumed to be only retardation of thought processes, then the entire problem will be suppressed, with serious consequences for both the patients and their families. Importantly, the non-pharmacological treatment of cognitive symptoms in patients with Parkinson's disease has attracted increasing interest and has even been included in treatment guidelines, such as cognitive training and physical interventions. Furthermore, as with Alzheimer's disease, multidomain interventions that combine cognitive training with physical interventions, nutritional guidance, social activity, sleep hygiene, and management of relevant health issues (e.g., cardiovascular disease and hearing impairment) will be important in the future for the prevention of cognitive decline in people with Parkinson's disease, too.

With this Research Topic, we hope to spark a wide-ranging debate to make progress in recognizing, diagnosing, treating, and, ultimately, preventing cognitive dysfunction in people with Parkinson's disease in all of its many aspects. We hope to do more than pinpoint our present status quo and hope to motivate our colleagues to conduct further research and explore promising ideas for the future.

